# Decent Living Standards: Material Prerequisites for Human Wellbeing

**DOI:** 10.1007/s11205-017-1650-0

**Published:** 2017-05-23

**Authors:** Narasimha D. Rao, Jihoon Min

**Affiliations:** International Institute for Applied Systems Analysis (IIASA), Schlossplatz 1, 2361 Laxenburg, Austria

**Keywords:** Decent living, Multidimensional Poverty, Human wellbeing, Human development, Reference budget, Fair wage

## Abstract

We define a set of universal, irreducible and essential set of material conditions for achieving basic human wellbeing, along with indicators and quantitative thresholds, which can be operationalized for societies based on local customs and preferences. We draw support for this decent living standard (DLS) from different accounts of basic justice, including the capability approach and basic needs. The DLS goes beyond existing multidimensional poverty indicators by comprehensively addressing living conditions and the means of social participation. The DLS offers a normative basis to develop minimum wage and reference budgets, and to assess the environmental impacts, such as climate change, of eradicating poverty.

**Electronic supplementary material:**

The online version of this article (doi:10.1007/s11205-017-1650-0) contains supplementary material, which is available to authorized users.

## Introduction

What, concretely, are the essential constituents of a *decent* life—one that goes beyond just subsistence, or ‘extreme poverty’? What ‘things’ should people have, and what resources do societies need to provide these goods? The limitations of income, and particularly the International Poverty Line, as a measure of poverty are now well understood (Reddy and Pogge 2009; Reddy 2008; Stiglitz et al. 2009). Without some notion of human requirements, there is no coherent way to specify an income level, let alone across countries, that can support a particular standard of living. Since the formulation of the Human Development Index (HDI) published in the UN Human Development Report in 1990, a number of multidimensional indicators of poverty have been proposed, such as the Multidimensional Poverty Index (MPI), and, more recently, the Social Progress Indicator (SPI). Generally, the focus of these indicators is on measuring outcomes of human wellbeing, rather than on specifying the requirements for achieving these outcomes. Here, we propose a set of *material* requirements that are essential for human flourishing, or what we call decent living standards (“DLS”). The DLS aims to be a starting point for comprehensively specifying the material constituents of a multidimensional poverty indicator, such as the SPI. Another motivation for focusing on material conditions is to provide a basis for determining the dependence of poverty eradication on natural resources and guiding their allocation where relevant.^1^

Our proposed DLS rests on previous conceptualizations of poverty and basic justice. Specifically, we draw from previous basic needs approaches (Doyal and Gough 1991; Max-Neef et al. 1991; Wiggins 1998), but also find support for the DLS from the capabilities approach (Nussbaum 2000; Sen 1987, 1993), as we elaborate later. Proponents of both approaches define basic needs (or capabilities) at a level of abstraction that eschews the specification of material commodities, in part due to their dependence on peoples’ culture, context and physical characteristics. The basic needs approaches of Max-Neef and Doyal & Gough (D&G) do grapple with the material dependence of human needs (material ‘satisfiers’, in their parlance). However, neither approach goes so far as to specify a universal set of material satisfiers of basic human needs. We fill this gap here. We see the DLS as a set of material conditions that people everywhere ought to have, no matter what their intentions or conception of a good life, or what other rights they may claim. These material requirements have no intrinsic value of their own. They are justified as entitlements only to the extent they are essential preconditions to meet basic needs or provide central capabilities. We specify the extent to which and how such material conditions can be generalized and specified for everyone, and where democratic processes would have to take over to reach the level of specificity required for their full operationalization. We argue, using global survey data, that in some cases particular commodities deserve inclusion in the DLS, where people globally reveal an overwhelming proclivity for them other potential alternatives. We also show that the provision of DLS to households generates further social (material) prerequisites at various scales, including at the community- and societal level.

The rest of the paper is organized as follows. In Sect. 2, we discuss the support for DLS in basic needs and capability theories, in resourcist approaches to justice, and in international law and policy. In Sect. 3, we elaborate on the concept of DLS and its scope, and propose some principles that are necessary to guide the selection and specification of its components. In Sect. 4, we discuss the practical applications of such a DLS, comparing it to other poverty indicators, and to reference budgets/living wage estimations. In Sect. 5, we lay out the actual components of the DLS, the rationale for their inclusion, and quantity threshold indicators, where relevant. In Sect. 6, we conclude with some thoughts for further work.

## Theoretical Background

Precedence for defining a DLS lies in philosophy and international law and policy. The former provides an ethical basis from which to comprehensively define a DLS, while the latter lends support to defining a universal standard in terms of living conditions and doing so as a matter of human rights.

### Decent Living Standards as a Matter of Justice

Several streams of thought support the notion that people ought to be entitled to, no matter what else they want, an inviolable set of goods (a “basic minimum”), to flourish in a just society. These include notions of primary goods (Rawls 1971), basic goods (Reinert 2011), the basic needs approaches mentioned above, rights to decent standards of living (Blake 2001), and arguably even the capabilities approaches (Nussbaum 2000; Sen 1987). The capabilities approaches in principle define poverty in terms of lack of choices to carry out various functions. Nussbaum lends support to the importance of certain *central* human capabilities, which are universal entitlements, regardless of people’s relative status in society, or of other values they hold (Nussbaum 2000). These central capabilities (life; bodily health; bodily integrity; senses, imagination, and thought; emotions; practical reason; affiliation; other species; play; and control over one’s environment) provide a basis to define universal material requirements for human flourishing, if it can be established that these requirements are instrumental and essential. In contrast to Nussbaum, Sen is known to place “doing” and “being” above “having” in defining standards for living (Sen 1987). Sen’s principle objection is that people have different abilities to convert resources into functioning. However, despite Sen’s reluctance to privilege possessions, he acknowledges (but does not extensively engage with) the idea that some basic capabilities may be amenable to commodification, delineating them into those that may not vary much across people (such as meeting nutritional requirements, escaping avoidable disease, being educated, and being sheltered) and those that may depend significantly on culture (such as avoiding shame, participating in community activities, and having self-respect) (Alkire 2002:186). Taking these together, it is possible to draw a common thread of capabilities between Nussbaum and Sen that provide opportunities for good health and security (“physical wellbeing”, as we later refer), which lend themselves relatively easily to defining essential material requirements. Another commonality pertains to the importance of social engagement, which may be interpreted as not just engagement with people (“affiliation” and “participating in community activities”), but also critical engagement with knowledge about the world (“being educated”, “practical reason”,“other species”). We consider these capabilities as enabling “social wellbeing”. While these capabilities are more culturally specific and difficult to relate to commodities, we later justify the inclusion of only the *means* of social engagement in a DLS. Other capabilities relate to human dignity and psychological wellbeing, which we do not see as reliant on material conditions.^2^

As we aim to do in this study, there have been several attempts to develop social indicators in the past, some that explicitly aim to operationalize capabilities or others that resemble them (Robeyns 2006). The HDI is the most well-known case of the former. Ramos and Silber (2005) use empirical analysis to compare several multidimensional human development approaches, and show that there is a great empirical resemblance between them. Robeyns points out that the capability approach “offers the underpinnings of a multidimensional empirical analysis”, and a basis to integrate theory and practice. Among indicators that bear resemblance to capabilities, the most similar to our effort is the Dutch index of living conditions (Boelhouwer 2002), which has many common dimensions, but is more of a positive than a normative indicator, and doesn’t focus on material resources.

Proponents of basic needs approaches, particularly Max-Neef and D&G, more directly justify a material basis for a ‘basic minimum’, through the notion of *satisfiers of,* or *intermediate,* needs, which are essential preconditions to meet basic needs. Both Max-Neef and D&G delineate universal satisfiers from context-specific satisfiers in principle, but they give limited attention to concretely defining universal satisfiers. D&G define all intermediate needs as having to fulfill the requirement that their lack can lead to a sustained degradation of people’s basic human needs, which they define as physical health and critical autonomy.^3^ Briefly, sound physical heath is interpreted as freedom from chronic disability, disease, and impairment of cognitive function. Autonomy reflects the ability to learn, work, engage in and reflect on culture, and enjoy leisure. Wiggins (1998) also describes *absolute* needs as having to meet the test of being necessary and sufficient to avoid serious harm. We see D&G’s categories of physical health and autonomy as directly parallel to the physical and social wellbeing related capabilities described above. Furthermore, the notion of harm avoidance is helpful to identify risks to wellbeing and the material conditions that can mitigate them.

**Fig. 1:**
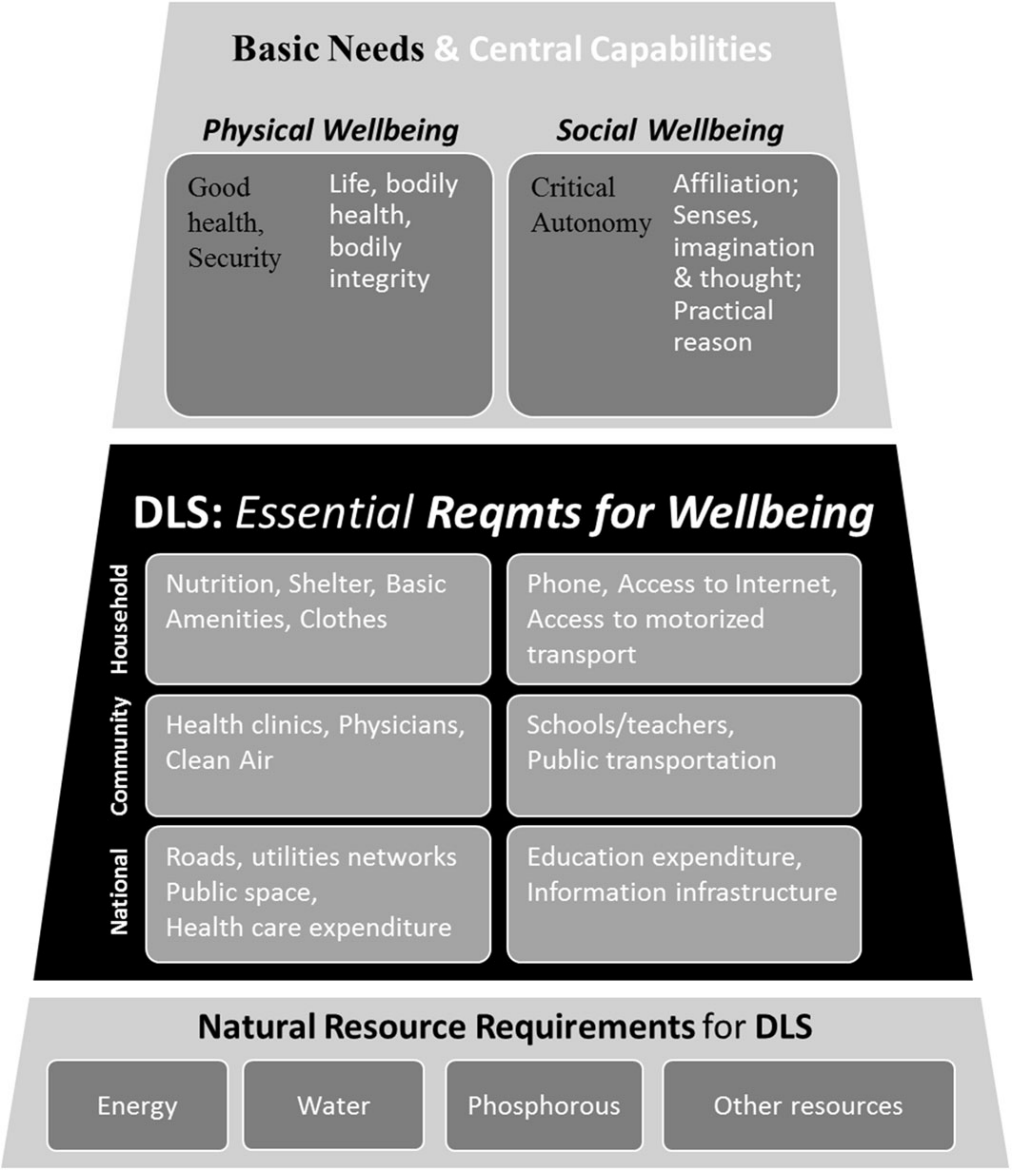
Decent living standards (DLS): hierarchy of material requirements and their derivation. We use the language of Doyal and Gough (1991) for basic needs and Nussbaum (2000) for central capabilities to define physical and social wellbeing, for which the DLS serve as prerequisites

One way to interpret the DLS is as a deepening of the hierarchy of intermediate needs or satisfiers, so as to make the notion of basic needs or capabilities operational—to identify what universal material satisfiers are required by people *everywhere*; which, in turn, inform what material resources in countries are required to provide those satisfiers. Figure 1 displays this hierarchy of material requirements, deriving principally from basic needs and capabilities, and enabling physical and social wellbeing.

### Decent Living Standards as a Basis for Resource Allocation

The DLS is also intended to form the basis for determining the energy and other resource requirements to eradicate poverty (Rao and Baer 2012). This view has precedence in political philosophy. Some have viewed basic human rights as giving rise to resource entitlements (Dworkin 1981; Pogge 2002), while others also view rights to decent living as providing a backstop against the burdens of environmental harm (Caney 2009, 2010), or an important dimension of distributive justice (Sovacool and Dworkin 2015). Walker et al. (2016) use the Minimum Income Standard (MIS) to justify a minimum energy requirement for the UK, by identifying the energy (service) dependence of identified commodities. These energy needs, when defined based on a universal minimum living standard, but country-specific resource conditions, also provides a basis to assess the adequacy of carbon space in a climate-constrained world to allow for this minimum living standard globally under different scenarios of future low-carbon technology development (Lamb and Rao 2015). For instance, if energy for poverty eradication were limited even in the most optimistic scenarios of technological achievement, the moral obligation to reduce emissions (from non-essential consumption) in industrialized countries would be intensified. On the other hand, if energy requirements for poverty eradication were modest in comparison to expected energy growth in developing countries alone, equitable allocation of climate mitigation efforts may still be desirable, but not necessarily driven by the objective to yield carbon space for poverty eradication.

### Precedence in International Law and Policy

In the limited available guidance in international law and policy, a common aim seems to be to define a set of *living conditions* that provide more than subsistence, and possibly even more than the traditional poverty dimensions of nutrition, health and education. The Human Development Report in 1993 describes decent standard of living as “the capability of living a healthy life, guaranteeing physical and social mobility, communicating and participating in the life of the community (including consumption)” (UNDP 1993). The limited attention given to elaborating or justifying this definition is noteworthy, considering that having access to resources to achieve a decent living standard was the ostensible basis for including income in the HDI. Nevertheless, this definition includes many elements that go well beyond basic subsistence and security. In particular, the reference to *mobility, communication* and *participation* in society are noteworthy.

The International Covenant on Economic, Social and Cultural Rights (ICESCR) also embodies a broad conception of living standards.^4^ Article 11.1, defines a right to an “adequate standard of living…including adequate food, clothing and housing, and to the continuous improvement of living conditions”. The ICESCR includes other related rights, such as to participation, self-determination, life, among other conditions. However, as a political document, these covenants have more symbolic than practical or normative value. Nevertheless, having 164 parties and four signatories, they are indicative of widely held aspirations for people, which should not be taken lightly.

## Other Multidimensional Poverty and Minimum Wage Indicators

This DLS has parallels with other multidimensional poverty indicators, and with policy efforts to define minimum wages, which are intended to provide sufficient means to purchase a set of essential commodities (Anker 2006). It is useful to compare these to the DLS, since they share common goals related to operationalizing poverty definitions for policy. In comparison to DLS, the poverty indicators tend to focus more on outcomes, rather than on material prerequisites, and they tend to be context-specific, and not universal. We elaborate both comparisons below.

### Other Multidimensional poverty indicators

A number of multidimensional poverty indicators have been developed, including the Multidimensional Poverty Index (MPI),^5^ the Individual Deprivation Measure (IDM),^6^ and the Social Progress Index (SPI).^7^ All these indicators have some material components, but share the goal of measuring *outcomes* of various dimensions of human wellbeing. They provide different ways to more realistically track the progress of poverty and of societal wellbeing as a whole. However, they have important differences among them, in scope and approach. MPI focuses on households (thus ignoring intra-household dynamics). It covers education, health and six living conditions, the last of which are implicitly substitutable in how they are counted into the composite indicator. IDM focuses on individuals (gender), and covers a broad range of social and economic deprivations. Of most importance, it is derived entirely from participatory methods in multiple countries. The IDM selects 15 dimensions for its final poverty measure, after soliciting a ranking from participants of all dimensions of importance to them. SPI is a collective, national metric that is also broad in scope, based on the Fitoussi Commission’s report on welfare beyond GDP (Report of the Commission on the Measurement of Economic Performance and Social Progress). Of interest for this work is that by focusing on outcomes, they provide limited guidance on the means to overcome deprivations. In many cases, the outcomes are defined in terms of possessions (i.e., ‘having’ something), such as having adequate nutrition, or having a television. However, in some dimensions, such as health (e.g., life expectancy or pollution-related deaths in the SPI), it is not specified how these indicators translate into means.

The Dutch index of living conditions mentioned earlier also shares many dimensions with the DLS. However, it was developed to track the state of society over time, without any normative content about minimum essential living conditions.

The MPI, IDM and SPI all lend support to the DLS, to the extent that all the underlying dimensions of deprivations in the DLS are, at some level of abstraction, part of these indicators. The DLS is not as comprehensive as the IDM or the SPI in developing non-material dimensions, particularly those applicable to political and social rights. The DLS goes beyond these other poverty indicators, however, by focusing on means, or essential intermediate needs (“satisfiers”). In particular, the DLS accords living conditions greater importance and defines the means to engage with contemporary society in more detail (See Sect. 4.2).

### Reference Budgets and Living Wages

This project shares similarities with ongoing work on reference budgets and fair wages (Anker 2006; Deeming 2015). It is similar in that it seeks a high degree of specificity in measures of commodities in the DLS. However, other reference budget initiatives are all national (e.g., UK, US) or regional (e.g., EU). This project seeks to first define *global universal* satisfiers that transcend contextual dependence, then define context-specific thresholds or guidelines for such thresholds.

Another important departure from the reference budget initiatives is in the normative content. In national social policy in Europe and the United States, efforts to define fair wages or reference budgets for poverty are derived from the purchasing power of a broader set of goods and services (baskets) in different countries. However, these reference budgets in the US are narrowly defined based on food, or in Europe have evolved largely independent of each other, and with limited, if any, standardization or normative justification (Deeming 2015). This has only recently begun to change in Europe (Storms et al. 2014). Current efforts to enumerate minimum living standards in Europe tend to be national, and focus on participatory methods (Bradshaw et al. 2008; Deeming 2015). For instance, the Minimum Living Standard (MIS) in the UK was developed with participatory methods. What people characterize as needs reflect cultural embeddedness and comforts to which people have become accustomed.^8^ This brings into question the generalizability to other regions, particular developing countries.

## Decent Living Standards—Concepts and Principles

We propose the DLS as a ‘lowest common denominator’ of basic material requirements that are instrumental (but not sufficient) to achieve physical, and to an extent social, dimensions of human wellbeing, whether conceived as basic needs or basic capabilities, and independent of peoples’ values or relative stature in society.

In the case of social wellbeing, it is harder to conceptualize commodity dependence, particularly considering that material possessions may satisfy social goals (e.g., status symbols) due to prevailing norms, not because they are intrinsically essential (Max-Neef et al. 1991). As described further below, we restrict our excursion into the social realm only to the *means* of social engagement, not the fulfillment of any social goals. To the extent these are non-essential and value-driven, we argue they belong in a DLS only if they are globally desired by an overwhelming majority of people.

Below we describe in more detail a set of principles to guide the selection of commodities into the DLS. We then discuss the limitations to the scope of the DLS.

### Guiding Principles

In justifying a DLS in terms of material requirements for everyone, we face a number of challenges. The most obvious is that multiple commodities may satisfy a need or capability, and that the appropriate choice may be contingent on culture and other contextual factors. Further, we indicated earlier that harm avoidance, as used in the basic needs approaches, is a useful basis from which to develop selection criteria for commodities. However, harm is also contingent on context and on human vulnerabilities, and inadequately specified in basic needs theories. Third, since one of our objectives is to link poverty eradication to natural resource use, we need to be comprehensive in determining essential resource requirements. We introduce some principles to guide the specification of universal satisfiers in light of these three challenges.

1. *Constituents of a DLS must either be necessary and indispensable, or globally desired.* There may be many material satisfiers that can serve a need or capability. We therefore need criteria for inclusion into a DLS. A good belongs in a DLS if and only if it satisfies conditions (a) AND (b) AND (either c.1 or c.2) below:
  - It satisfies at least one basic need or capability (that is, it either helps fulfill a dimension, or prevents harm to people’s own fulfillment)^9^;
  - It doesn’t harm the fulfillment of anybody’s needs or capabilities;
  -
    1. It is the only satisfier of at least one basic need/capability;
    2. It is one of many competing satisfiers, but it is overwhelmingly preferred *at a global scale for at least one dimension*. The bar must be set high for such support—goods must be owned or desired by an overwhelming majority populations in *all* countries where they are available and affordable.

If a need/capability can be met by a number of satisfiers that don’t meet condition (c.1) or (c.2), then the DLS constituent has to be decided at an implementation phase, through participatory approaches.

Take some examples. For adequate nutrition, if many different diets (e.g., meat-based or vegetarian) can provide the required nutrition and none is universally desired [(a) and (b) met, but not (c.1) nor (c.2)], then the specification of a DLS should remain at the level of nutrients, allowing for different diets to be determined at the local level through participatory methods. On the other hand, consider education, which arguably depends on knowledge acquisition from media as well as from the classroom. If newspapers and televisions are two competing media that offer equivalent content, televisions may be part of a DLS if they are universally desired [violate (c.1) but meet (c.2)]. However, if alcohol were universally desired [(c.2) satisfied) and consumed, but known to cause harm to human health (violating (b)], it should not be included.

2. *A DLS limits the risk of harm to achieving basic human wellbeing to an acceptable threshold.* DLS constituents may be included because they prevent harm to meeting basic needs, such as good health. However, the assessment of potential harm is not straightforward. The ambiguity lies in at least two aspects: what is the risk of an effect (which in turn is the product of the severity of an effect and its likelihood); and one’s vulnerability to it. It is the combination of these that together define the risk of harm. Different resources would be required to mitigate risk depending on the extent of risk aversion one chooses, as is well known in risk analysis. Because of this dependence, a DLS eventually would need to define such risk thresholds, notably for different types of people, who have different levels of risk tolerance. For instance, the average person may tolerate a few days of extreme heat or muggy weather, particularly with adequate access to fluids and shade, but the elderly may have a much lower tolerance for the same conditions. A DLS in practice would be contingent on the establishment of such risk thresholds.

We propose two qualitative boundary conditions for setting these thresholds. On the one hand, everyone ought to be insulated from potentially fatal conditions, even of low likelihood (e.g., vaccinations against diseases, such as polio). Furthermore, harm should also include prolonged exposure to extreme discomfort. Freedom from ‘extreme discomfort’ in a household, for example, can be defined as freedom from *prolonged* exposure to indoor air pollution, inadequate lighting at night, high ambient temperatures or humidity, *excessive* labor to meet other basic needs (e.g., cooking or washing). Such extreme conditions can debilitate (physically, emotionally or psychologically). Excessive labor on household chores can be debilitating, but it can also reduce time available to pursue leisure or gainful activities. For example, women spend hours collecting and carrying firewood and water in poor countries, cooking, and washing clothes, which restricts their opportunities and choices for participating in other roles in society (Pachauri and Rao 2013). There is a judgment involved as to when the opportunity cost of her time becomes ‘harmful’, but at some point this opportunity cost must be recognized as an unjust encumbrance.

3. *Individual entitlements give rise to material requirements at the household, community or societal level*. The DLS are individual entitlements, but society is typically organized such that people share material resources, at different levels or aggregation. We find it useful to define DLE at three scales: household, community and society at large. Families share homes and utility access (e.g., electricity connections); members of a community typically share schools, hospitals, or transport infrastructure to achieve mobility. The provision of these facilities, in turn, may necessitate the development and use of physical infrastructure at a city, state or national level (e.g., road networks, electricity grids). The levels and types of sharing mechanisms are a function of our times, reflecting norms, technology, economics, or other societal characteristics. This has three practical implications for a DLS: different DLS constituents may be defined and measured at different levels of aggregation, in accordance with prevailing norms; these definitions may need to be revised in the future if changes in these norms necessitate different modes of organization for particular goods/services; the actual enjoyment of these DLS constituents depends on the equitable distribution of these constituents (even within households (see Sect. 4.2 below).

### Boundaries of Inquiry

*Focus on essential material needs* The scope of this project doesn’t allow a comprehensive assessment of a DLS, but rather focuses on essential material elements. Any DLS must include political, civil and psychological “goods” (whether they are considered to be rights, liberties, or other forms of entitlements), which enable people to have self-esteem, and engage as political constituents, namely to understand, participate in, and dissent against political institutions that govern them (Heinrichs 2006). We take these rights for granted, but limit their operationalization to aspects that *principally* entail *material* needs, namely the means of social engagement. For example, psychological wellbeing (e.g., self-esteem), once people have *other* elements of a DLS, such as good health and education, depends far less on material possessions than on how people treat each other. Political institutions and granting political rights do require physical infrastructure to function (e.g., voting infrastructure, national defense), however to our knowledge there is little basis to link ‘good’ institutions (e.g. democracy vs. autocracy) to the extent of infrastructure. We set this aside for further research. What non-material societal pre-conditions are necessary to ensure that political institutions provide *decent* political/social rights is a complex and deep question, which we do not have the scope to address. We refer readers to the IDM to learn more about what political and social rights matter to people, since it was developed based on participatory approaches.

*Focus on the definition of a DLS, not its realization* This paper sets out first principles towards defining a specific basket of goods and services for individuals in a particular society. Further steps that would need to be taken to fully define a DLS for a particular society and to take concrete steps towards their realization. These include several policy challenges, such as on whom the responsibility to provide DLS falls, how to make DLS constituents affordable, and to ensure equitable delivery of a DLS to all. These are important questions for subsequent research.

## Decent Living Standards—Constituents and Indicators

We now turn to the specific constituents of a DLS, the universal material satisfiers of basic human wellbeing (summarized in Table 1). We group them into satisfiers of physical and social wellbeing dimensions respectively. We then indicate the material requirements more specifically, delineated into those that are a property of a household and those that represent aggregate societal requirements, which would be shared at some level of social organization. We then follow with an explanation (rationale) of each item. We specify indicators and *minimum* quantities including any empirical support, where relevant and feasible. We also indicate where context-specific customizations (such as through participatory processes) would be appropriate. Some of the constituents and their quantitative thresholds have been introduced and justified in earlier work (Rao and Baer 2012). We make reference to the U.N's Sustainable Development Goals (SDG) where relevant.

### Nutrition

*Universal satisfiers* Adequate nutrition, including macronutrients (energy, protein) and micronutrients (including iron, zinc and vitamins); cold storage.

*Household requirements* Minimum daily (context-dependent) intake of total calories, protein, vitamins and minerals; a modest sized refrigerator (e.g., 100 l).

*Rationale* Nutritional requirements are a complex but well-trodden field of public health. It is well known that in many developing countries malnourishment (among the poor) and obesity (among the middle and higher income) are prevalent and growing (FAO 2008). This has contributed to health disparities in these countries (Hawkesworth et al. 2010). More recent evidence shows that micronutrient nourishment (specifically protein, iron and zinc) has declined from the pressure of increased agricultural production of high-yield cereals with lower nutritional content (DeFries et al. 2015). Thus, it is important not only to ensure adequate calories, but the right type of foods.

The actual daily requirements can be set at a national level. The Food and Agricultural Organization (FAO) supports the use of a reference set of calorie intake requirements for men and women, on the basis of which deviations can be calculated for differences in age, and activity level (FAO 2001). Many countries have public health institutions that publish dietary guidelines for total calorie intake, and in some cases for micronutrients.

Having cold storage avoids risks of ill health from food-borne diseases and discomfort related to the time spent preparing and purchasing food items. Women usually bear this burden, in addition to the tasks of collecting water and cooking fuel. The extent of discomfort is contingent on a number of factors, including climate and diet,^10^ but also access to markets. In many urban areas, where fresh food can be purchased on a daily basis, it is not easy to argue that refrigerators are universally essential, or that they always avoid *extreme* discomfort. However, given that the empirical support (see below) indicates an overwhelming desire to own a refrigerator, cold storage merit inclusion at least on the basis of being an overwhelmingly desired satisfier with no substitutes (See Sect. 1).

*Empirical Support* Almost 100% households own refrigerators in developed economies. In urban areas of select emerging economies (China, India, Brazil, South Africa), electricity access and refrigerator ownership has already, or is trending towards, saturation at over 90% penetration above a certain income threshold (See Table 3, in the Supplementary material).

**Table 1:** Decent living standards—material requirements indicators

Decent living standard dimensions	Household requirements	Collective requirements
*Physical wellbeing*		
Nutrition		
Food	Total calories, protein, micronutrients	
Cold storage	Fridge (or other technology)	
Shelter	Solid walls and roof	
Living conditions		
Sufficient, safe space Basic comfort (bounded temperature/humidity) Hygiene	Minimum floor space Modern heating/cooling equipment In-house improved toilets Minimum, accessible water supply	Electricity, water and sanitation infrastructure
Clothing	Minimum clothing materials	Washing machines per 1000 persons
Health care		
Accessible and adequate health care facilities		Minimum health expenditure per cap Minimum physicians per 1000 persons
Air quality		
Maximum ambient particulate matter (PM_2.5_)	Clean cook stoves	Restricted transport infrastructure
*Social wellbeing*		
Education		
Nine years schooling		Equipped schools Teachers per 1000 persons
Communication	Phone (1 per adult)	ICT infrastructure
Information access	Television/internet device	
Mobility	Access to public transport, or vehicle, if essential	Public transport and road infrastructure
Freedom to gather/dissent		Public space, sq. m. per 1000 persons

### Shelter

*Universal satisfier* Durable homes that are resilient to severe climate and disease-carrying vectors.

*Household requirements* Solid roof and walls: brick, wood, concrete, or cement/steel construction.

*Rationale* Safe shelter (SDG 11.1) is, like food, a universally accepted goal of development policy, and a component of multi-dimensional poverty indicators. However, its formulation equally widely lacks specificity.

The UN Habitat places sufficient space and durable housing as its main priority for moving people out of slums in urban areas.^11^ Sturdy construction protects from inclement weather, and therefore provides basic physical security.

### Living Conditions

*Universal satisfiers* (a) Minimum floor space; (b) adequate lighting; (c) basic comfort (bounded range of temperature and humidity in inhabited spaces); (d) adequate, accessible water supply; and (e) safe waste disposal.

*Household requirements* (a) Minimum of 30 m^2^ and 10 m^2^ per additional person, above three members; (b) electrical lighting (c) modern heating/cooling equipment, if necessary to remain within the comfort conditions^12^; (d) Adequate, reliable water supply (minimum of 50 L per capita per day) from an accessible water source^13^; (e) in-house improved toilets.^14^

*Collective requirements* The provision of the above household amenities may require the presence of a backbone infrastructure, for electricity, water and sanitation. The industrial organization and technology for this infrastructure depends on location and prevailing norms, and therefore need to be decided locally. For instance, today centralized electricity grids at a national scale provide electricity access, but water and sanitation typically fall within state- or municipal jurisdiction. The technology for sanitation may differ depending on cultural norms.

*Rationale* Overcrowding can lead to a number of health risks (e.g., related to sanitation), and less visible emotional stresses from lack of privacy and personal freedom. The amount of sufficient space should be decided at a local level. However, as a guide, it is worth considering national guidelines for minimum living space in affluent, but densely populated, countries. For instance, in Taiwan, recommended minimum living space ranges from 7 to 13 m^2^ per person, depending on number of members. In Korea, the minimum standard is 12 m^2^ for one person, and 8–10 m^2^ for each additional member. In previous work, we suggest this threshold should be closer to 10 m^2^/cap, which is the actual floor space to which middle class Indian homes plateau (Rao and Baer 2012). We additionally consider that homes have shared spaces—bathrooms and kitchen—that don’t scale with household size, but necessitate a minimum floor space. China’s average home size urban (rural) areas of ~32 (37) m^2^ offers another potential benchmark,^15^ since families are typically small (due to the historical one-child policy), and living standards on average in China are likely to reflect an aggregation of a broad range of population densities and living conditions.

The lighting and space conditioning standards speak to habitability, and the avoidance of extreme conditions that may cause extreme discomfort or, in the worst case, death. The risk of these outcomes would vary with the severity of climatic conditions and with people’s vulnerability (e.g., elderly may have lower tolerance than youth). Similar to nutrition, further thresholds of exposure (e.g., maximum degree-days outside the comfort zone, or humidity levels) and vulnerability would have to be established for countries based on average population group characteristics and climatic conditions. There are many available references for defining a comfort zone, such as national guidelines on workplace occupancy conditions (e.g., US ASHRAE 55).^16^ These can be adjusted for peoples’ adaptive preferences in different climatic conditions (Nicol 2004).

Water supply and sanitation, like food, have been examined extensively in public health and development policy. Gleick (1998) suggests that 50 l per capita per day is a minimum for all human ablutions. The World Bank has indicators for both improved water and sanitation, which provide useful guides for the quality and accessibility of these services. We adopt the World Bank’s indicator for improved sanitation and water source. In-house or accessible water supply obviates hours of labor that typically women undertake to collect water. Improved and accessible sanitation is essential not only to avoid the spread of disease from open defecation, but also to provide safe conditions for women.

### Clothing

*Universal satisfier* (a) Sufficient clothing to achieve basic comfort (as defined above) in prevailing climatic conditions; (b) access to washing machines.

*Household requirements* A certain amount of cloth (m^2^) with adequate materials catered to local climate;

*Collective requirements* Minimum number of shared washing machines per 1000.

*Rationale* As with food and shelter, clothing is to our knowledge an integral element of all poverty indicators, but also relatively unspecified. Clothing is also a feature of human life that is deeply embedded in culture and tradition. This makes it a clear candidate for further specification through local participatory methods. The only feature of normative importance is that these clothing are sufficient for daily activity in local climatic conditions.

Washing clothes is essential for basic hygiene. The need for washing machines is a matter of avoiding extreme discomfort from excessive manual labor. However, washing machines may be shared by number of households. In urban areas, shared facilities in apartment buildings and communities is already common practice. In rural areas, where homes are much more dispersed, sharing facilities can become a nuisance. However, since we aim to cater to the norm, not the exception, we eschew individual household entitlements to washing machines.

*Empirical Support* In most developed countries, most households have washing machines. However, communal washing facilities are common in urban areas of many countries, including the United States, where only 82% of homes have washing machines (Table 3, in the Supplementary material).

### Health Care

*Universal satisfier* Sufficient and accessible preventive and curative health care facilities.

*Collective requirements* Minimum physicians per 1000 people (possible range of 1.5–1.7); and minimum national health expenditure (possible range of PPP^17^$~450–700 per cap).

*Rationale* Typical health outcomes in poverty indicators, such as life expectancy and infant mortality, offer little insight on the needs for health care. Although good health depends first on adequate nutrition and hygienic conditions, in reality, humans inevitably face disease, accidents, and other health hazards. Medical care is critical to prevent disease (e.g., vaccines), provide child care, and provide basic curative care. In order to provide these basic services, there needs to be sufficient health posts within reach of the population, with adequate facilities in each (e.g., cooling for medicines, electricity for X-rays) and qualified staff. These conditions are by no means sufficient to ensure a high quality of health care, but can be considered necessary.

But how should a minimum set of material conditions be determined? Health care services are necessary to reduce morbidity, avoid premature death and care for the elderly (palliative care) as they lose functioning capability. All these characteristics of a healthy society are well represented by average life expectancy, which is the primary measure of health in poverty measures, such as the HDI, and the more recent SPI. There is indeed a positive relationship between the resources committed to a health care system and average life expectancy, albeit with significant variation, and with diminishing returns beyond a point (See Supplementary material). This suggests that defining a DLS requires selecting a threshold for life expectancy. There is, however, no known normative basis to define a minimum length of a life.^18^ Subjective preference isn’t useful either, because people generally aspire to live longer. Rather than seeking a normative threshold, we instead select this threshold based on where empirically we find that resources cease to have a positive effect on life expectancy. Based on extensive empirical analysis of the correlation between life expectancy and a number of different indicators of health care resources, including per capita national expenditure, we find that health care expenditure is correlated with life expectancy (LE) (see Supplementary material) in a certain range, ~70–75 years, but not very much below (where improvements in LE require few resources) or above (where increasing health care system resources has little effect on improving LE).

On this basis, we propose that societies require a minimum health expenditure, to sustain average life expectancy of 70–75 years. The suggested expenditure per capita (and reference life expectancy) is only a guide—individual societies may customize this value based on specific features of their health care system. We also found that the number of physicians (specifically, ~1.5–1.7 per 1000) also correlates to life expectancy. However, since the number of physicians doesn’t raise particular material requirements (doctors don’t eat more calories than people of other professions), we focus on health care sector expenditure as the primary metric for a DLS.

*Empirical Support* We estimate an annual expenditure of $450–700 per capita, corresponding to the average cost of the more efficient half of countries that have achieved a life expectancy of >65 years (and infant mortality of <15 deaths per 1000 live births) and >74 years (and infant mortality of <25). One caveat, however, is that it is unclear to what extent these expenditures include preventive health care or whether the latter correlates with overall health care costs. See Supplementary material for details.

### Air Quality

*Universal satisfiers* Maximum particulate matter (PM) concentration^19^; This is a unique satisfier, since it is the restriction of a ‘bad’ material—particulate matter—which is a by-product of other commodities, including some that may be part of a DLS. This requirement, therefore, constrains the technologies used to meet other DLS.

*Household requirements* Modern cook stove, using gaseous fuel or electricity; modern heating/cooling equipment. Having a clean environment as part of decent living is echoed in D&G’s intermediate needs, and in the SPI and IDM indicators, but without further elaboration. According to the Global Burden of Disease, household air pollution (typically from burning biomass) is the third highest health risk factor, leading to over 4 million premature deaths per year (Lim et al. 2012), who are mainly women and children. Its avoidance requires that homes cook stoves and heating equipment run on liquid or gaseous fuels, rather than burn solid (biomass) fuels.

*Collective requirements* Ambient air pollution from other sources, including industry and transport, also contribute to health risks. This implies that the transport choices offered as part of mobility may have to include public transport in urban areas, and possibly even restrict engines to electric and other non-polluting technologies. The extent of these restrictions would be highly context specific, and therefore have to be determined at the local level.

### Education

*Universal satisfier* Adequate schooling with adequate facilities and staff.

*Collective requirements* Adequate number of schools, equipped with space, teaching staff, facilities, and balanced curriculum.

*Rationale* The human interest in gaining knowledge and the need for compulsory education is well established, and included in all mentioned poverty indicators. The duration of required schooling is more ambiguous. With regard to the duration, most countries (69%) that have minimum requirements require between 9 and 12 years, while 21% require only primary schooling.^20^ We choose the lower bound of the majority option for the DLS.

Quality of education is, however, difficult to measure. Unlike with health care, there isn’t a clear relationship between educational attainment (or teacher absenteeism) and education spending. These factors are set aside for future research.

### Information and Communication

*Universal satisfier* Household access to information and communication services.

*Household requirement* One phone per household, one television/computer monitor per household;

*Collective requirement* Accessible communication and television/internet infrastructure.

*Rationale* The importance of social and political engagement for human flourishing is found in all accounts of basic justice (Alkire 2002), and even in international human rights, as discussed earlier. Information services provide knowledge about society that enables people to critically engage as political participants.^21^ Access to information can even be considered part of learning, when more broadly construed as the acquisition of knowledge about the world and society.^22^ Such knowledge cannot be individually acquired without access to information services. The IDM and SPI include phone and internet access.

Technology plays a strong part in determining the medium of such access. As such, the specific satisfiers of this constituent of DLS is very much a product of the current times, and of our foreseeable future. For instance, it can be argued that people need have only newspapers for information. There is an element of conformity to globalized consumptive patterns inherent in the choice of cell phones and devices to access the Internet. However, these new technologies may indeed become essential to access these types of services, because they render older technologies obsolete and unavailable. Furthermore, even if alternatives do not die out, they are not able to provide the same level of access to information, which would lead to significant disparities in access to information, and therefore unequal enjoyment of basic rights to participation as equals in society.

Access to communication services is distinct from access to information, in that it entails the use of devices that enable interactive communication with other people, which is important for people to feel a sense of belonging and membership in community.

*Empirical Support* Almost 100% households own TVs and phones in developed economies. In urban areas of select emerging economies (China, India, Brazil, South Africa), ownership has already, or is trending towards, saturation at over 90% penetration above a certain income threshold.

### Mobility

*Universal satisfier* Access to adequate mobility options. ‘Adequate’ refers to the availability (within a certain distance from home) of motorized transport. Notably, adequate mobility can be provided with public transport. There may be exceptions in rural areas, which would have to be determined at a local level.

*Collective requirements* Adequate public transit in urban areas and road infrastructure to support access to paved road and motorized transport for all. In sparsely populated remote areas only, household ownership of vehicles may be necessary.

*Rationale* The importance of transport is understated in the literature. The MPI includes a vehicle, but only among a list of substitutable assets that comprise a living standard indicator. People universally have to either work away from their homes or access markets to sell wares for their livelihood. There is some evidence that through history people spend roughly the same amount of time on average (~1 h/day) traveling (Schafer and Victor 2000). It is just the mode of transport, and therefore the accessible distance, that has increased over time. If this is a fact, spending more time on traveling arguably can be construed as burdensome (and hence extreme discomfort). Without motorized transport of any kind, people’s lives would then be restricted to within a few kilometers of their home, which may lead to social exclusion, and restrict opportunities to participate in society, by way of selling wares, traveling for leisure, or learning about other societies.

The quantity of infrastructure that is required to provide everyone access is as far as we know an unaddressed research question. Future empirical investigation in this direction is necessary.

*Empirical Support* In developed countries, car ownership is often <85% and decreasing in urban areas. Vehicle ownership is consistently higher in rural areas, likely due to lack of alternatives.

### Freedom to Gather/Dissent

*Universal satisfier* Adequate and safely accessible public spaces.

*Collective requirements* Minimum public space per 1000 inhabitants (with adequate facilities to ensure safety, such as lighting at night).

*Rationale* Adequate public space prevents overcrowding, and is important to foster a sense of freedom, for the pursuit leisure activities, and to congregate for political and social activities. This is particularly important in densely populated urban areas. This is also an SDG (11.7), which emphasizes the need for such spaces for women, children, elderly and disabled people. Here too, there is no guidance available in literature for the amount of space. However, there should be ample empirical evidence from which to develop reasonable benchmarks in further research.

## Conclusions and Further Research

We have proposed a universal set of material commodities and conditions that households and societies require, at a minimum, for overcoming poverty and supporting a decent life for all. We go beyond existing indicators, both in scope and specificity. Hunger is not just adequate calories, but adequate vitamins and minerals. Shelter should have adequate space, solid construction, modern stoves, heating/cooling equipment, lighting, water and toilets, access to the Internet, and to public transportation. Communities should have schools and health clinics. Countries in turn should expend sufficient resources on physical infrastructure, health care and education to ensure the provision of these goods and services. None of these systems should generate air pollution beyond safe levels. Quantities of these items would to be specified locally, based on participatory methods, and further analysis. These DLS are also a function of our times—they have been specified based on current technologies and norms, but with care to including only those that have demonstrable universal appeal.

Nothing we propose is conceptually new—at a higher level of abstraction, the elements of the DLS can be traced to basic needs or capability theories. We have pushed the boundaries of specificity, so as to generate a dashboard for material poverty that is universal, but must be translated into quantities based on context and democratic processes. The DLS can guide the establishment of reference budgets and living wages, and development policies. They are also intended to identify the environmental resource requirements to provide a basic living standard to all, so as to assess whether there any conflicts between social and environmental sustainability at a global scale.

These requirements are not, however, sufficient to ensure wellbeing, nor do they necessarily overcome relative poverty. In societies with significant disparities and significant affluence among a few, people may be entitled to more, even if they have enough to avoid absolute deprivation. The realization of these goals raises another set of issues, not least is to make these services affordable.

## Electronic supplementary material

Below is the link to the electronic supplementary material.

Supplementary material 1 (docx 250  kb)

## Footnotes

1 We refer to dependence on resources through consumptive dimensions of human wellbeing, not through livelihoods. See Sect. 2.2.

2 This grouping of wellbeing into physical, social and psychological wellbeing is inspired by Narayan et al. (2000), who defines the following dimensions of wellbeing: material wellbeing, bodily wellbeing, social wellbeing, psychological wellbeing and freedom of choice.

3 D&G go further than other basic needs proponents and specify categories of intermediate (or essential) needs: nutritional food and water; protective housing; non-hazardous work and physical environment; appropriate health care; security in childhood; significant primary relationships; economic and physical security; appropriate education; and safe birth control and child-bearing. Doyal and Gough (1991).

4 International Covenant on Economics, Social and Cultural Rights, www.ohchr.org.

5 See Alkire and Santos (2014); www.ophi.org.uk.

6 See Wisor et al. (2015); www.iwda.org.au/introducing-the-individual-deprivation-measure/.

7 Social Progress Imperative (www.socialprogressimperative.org).

8 In the UK, repeated Minimum Income Standard (MIS) surveys show that people, particularly youth, include vacuums and irons in their list of ‘essential’ commodities as means to present themselves and their homes with adequate tidiness to visitors (Walker et al. 2016).

9 The notion that satisfiers can fulfill multiple needs is addressed in Max-Neef’s ‘synergistic’ satisfiers (Max-Neef et al. 1991).

10 Certain rural societies in China do not own refrigerators because they eat smoked meat and don’t consume diary. See http://www.theatlantic.com/technology/archive/2016/05/why-refrigerators-were-so-slow-to-catch-on-in-china/481029/.

11 See UN Habitat, “The Right to Adequate Housing”, Fact Sheet 21.

12 The importance of this threshold is to establish the *need* for air conditioning in hot and humid conditions, where no adaptation or technology can bring a home to within the comfort conditions.

13 World bank defines an accessible and improved drinking water source as: includes piped water on premises (piped household water connection located inside the user’s dwelling, plot or yard), and other improved drinking water sources (public taps or standpipes, tube wells or boreholes, protected dug wells, protected springs, and rainwater collection). World Development Indicators (http://data.worldbank.org/data-catalog/world-development-indicators).

14 Improved toilets are defined by the World Bank as flush toilets connected to a sewer system, septic tank, pit latrines or composting toilets. World Development Indicators.

15 See China National Statistics at http://www.stats.gov.cn/tjsj/ndsj/2014/zk/html/Z0617E.htm.

16 See https://www.ashrae.org/resources--publications/bookstore/standard-55.

17 This is a health sector-specific purchasing power parity (PPP) index provided by the World Bank.

18 There is research to indicate that age may even be the wrong metric—cognitive ability, rather than years, is a better indicator of aging (Skirbekk et al. 2012), which is consistent with the capabilities approach.

19 The WHO air quality guidelines (AQG) indicate annual mean levels of 10–35 µg/m^3^.

20 See UNESCO Education 2030. http://www.uis.unesco.org/Education/Pages/right-to-education-2030.aspx.

21 There would need to be conditions on the quality of information services (e.g., government propaganda alone would not meet the objective of providing people with adequately information to politically engage and critique government. However, this is set aside on the basis of not being a resource issue.

22 Doyal and Gough (1991) refer to the importance of cross-cultural knowledge as part of ‘critical’ autonomy, to be able to be self-reflective about one’s own culture.
