# Supplementary material for: Decent Living Standards: Material Prerequisites for Human Wellbeing
Source: Soc Indic Res. 2017 May 23;138(1):225–44. doi: 10.1007/s11205-017-1650-0 (PMC6013539; doi:10.1007/s11205-017-1650-0)
Supplement: Supplementary file 1 — Supplementary material 1 (docx 250 kb) [file 11205_2017_1650_MOESM1_ESM.docx]

# Appendix

*Health Care analysis*

Life expectancy is widely used to indicate the overall health status of a society. For example, the UN’s Human Development Index uses normalized life expectancy for the health dimension of the index (UNDP 2015). Child mortality, while partly reflected in life expectancy, is also an important indicator which is more directly linked to poverty and health service quality, and thus is considered as a universal development goal, which is exemplified in the health dimension of the Global Multidimensional Poverty Index (MPI) (Alkire & Santos 2014).

We normalize and combine life expectancy at birth and under-5 mortality rate (probability of dying by age 5 per 1000 live births) as one metric (hereinafter called “health status index”) for each country. Then we relate this metric to potential explanatory variables in each country and select ones with the highest explanatory power for our analysis. Potential explanatory factors we tested were:

1. Infrastructure
   1. Number of hospitals or other health centers per population
   2. Number of equipment (i.e. bed, CT, MRI) per population
2. Workforce
   1. Number of health care professionals (i.e. physicians, nurses, dentists, pharmacist) per population
3. Financing (expenditure)
   1. Total expenditure on health per capita (PPP $)
   2. Government expenditure on health per capita (PPP $)
4. Access to medicines
   1. Availability of 14 selected generic medicines in a sample of health facilities

The source for these statistics is Global Health Observatory (WHO 2016). We mainly use data from the year 2012, but when it is unavailable for the year, we use the most recent year with data.

Among these, we find ‘total expenditure on health per capita’ and ‘number of physicians per population’ independently explain variations in the health status index most significantly. The relationships between these factors are shown in Figure 1. Then, Figure 1a and Figure 1b are combined to generate Figure 2.

In Figure 2, we observe that a larger health expenditure is related to higher life expectancy and lower child mortality and that most countries with adequate expenditures cluster between certain levels of outcome (Figure 2b).

We set levels of life expectancy or child mortality that represent decent quality of life. One target of the UN Sustainable Development Goals is to reduce under-5 mortality to below 25. Figure 2b shows that all countries currently meeting this goal have life expectancy higher than 65. We classify this group of countries as *minimum-performance*.

In addition, we classify another group of countries with higher performance based on the figures. We observe the vertical distributions of the points in Figure 1a and Figure 1b converge at around the expenditure level of $1500. At that point, the minimum life expectancy reaches about 74-75, and the maximum under-5 mortality reaches around 15. We categorize countries that exceed these levels as *decent-performance.* This threshold for life expectancy is also supported by another study on ethical poverty lines (Edward 2006).

Both groups of countries (*minimum-* and *decent-performance*) exhibit wide ranges of health expenditures and numbers of physicians. So within each group, we provide summaries in Table 2 for 1) highly efficient countries (the efficient half in each distribution) and 2) all countries in the group. We can base our decent living energy analysis on representive values from each of these distributions.

Figure 1. Relationship between indicators for health status and two explanatory variables, health expenditure per capita per year, physicians per 1000


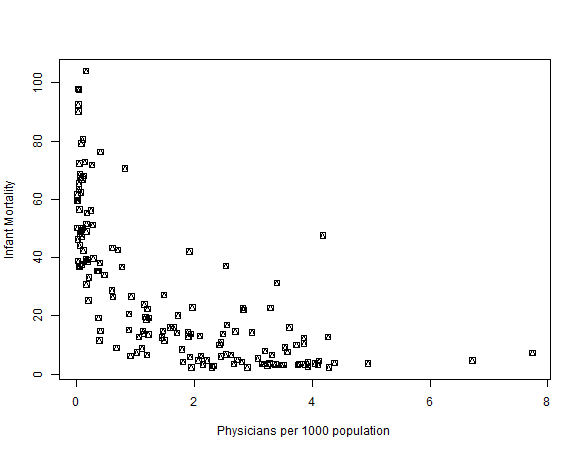

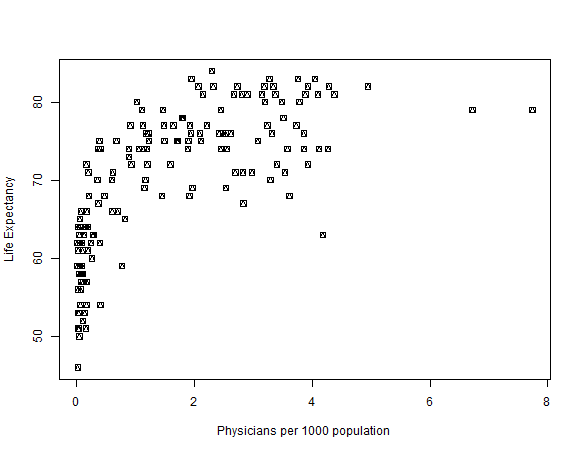


(a)

(b)

(c)

(d)


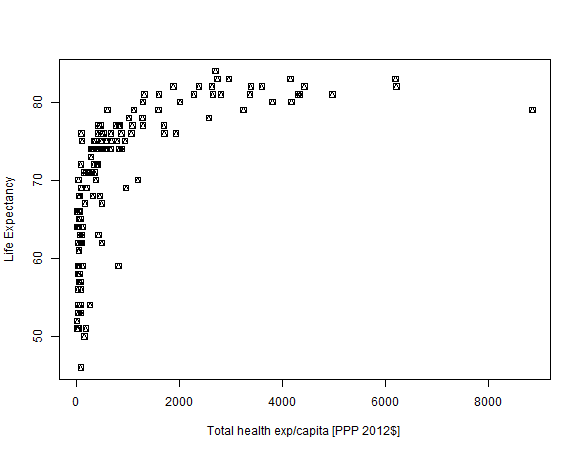

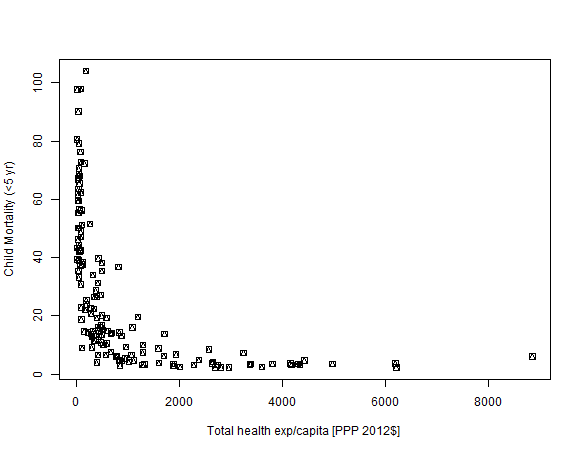

(a)

(b)

Figure 2. Relationship among life expectancy, child mortality, and health expenditure. The inset in (a) is enlarged and shown in (b). The two rectangles show the two performance groups. Colors represent the relative levels of the metric combining the two axes.

Table 2. Summary of minimum- and decent-performance groups. (a) Total health expenditure per capita (PPP $); (b) number of physicians per 1000 population.

| (PPP US$) |  | num.country | min  (a) | max | median | mean | std.dev |
| --- | --- | --- | --- | --- | --- | --- | --- |
| *minimum- performance* | All | 85 | 91 | 8845 | 883 | 1591 | 1656 |
|  | Eff. half ^a^ | 42 | 91 | 873 | 429 | 451 | 216 |
| *decent- performance* | All | 64 | 109 | 8845 | 1303 | 1972 | 1741 |
|  | Eff. half | 32 | 109 | 1290 | 665 | 687 | 315 |

^a^ ‘Efficient half’ means the countries ranked in the lower 50% according to total health expenditure per capita in each group.

| (persons) |  | num.country ^c^ | min  (b) | max | median | mean | std.dev |
| --- | --- | --- | --- | --- | --- | --- | --- |
| *minimum- performance* | All | 83 | 0.4 | 7.7 | 2.5 | 2.6 | 1.3 |
|  | Eff. half ^b^ | 42 | 0.4 | 2.5 | 1.5 | 1.5 | 0.6 |
| *decent- performance* | All | 63 | 0.4 | 7.7 | 2.5 | 2.7 | 1.4 |
|  | Eff. half | 32 | 0.4 | 2.5 | 1.9 | 1.7 | 0.6 |

^b^ ‘Efficient half’ means the countries ranked in the lower 50% according to the number of physicians per 1000 population in each group.

^c^ The number of countries are different between two tables because some countries do not have the information on the number of physicians.

## Asset Ownership

Table A3: Household appliance penetration in select industrialized and emerging economies, various years (2009-12). Sources: National statistics, Statista 2014, Euromonitor 2009, Demographic and Health Surveys, National household consumption and expenditure surveys. Income in per capita $2010PPP.

| Country | Income | Electricity Access | Television | Mobile phone | Refrigerator | Washing machines |
| --- | --- | --- | --- | --- | --- | --- |
| US | 48,374 | 100 | 98.7 | 93 | 99.8 | 82 |
| UK | 35,855 | 100 | 100 | 92 | 100 | 97 |
| Germany | 39,612 | 100 | 100 | >90 | 99 | 96 |
| France | 35,867 | 100 | 100 | 89 | 100 | 100 |
| Japan | 33,741 | 100 | 100 | 93 | 100 | 100 |
| Albania | 9,298 | 100 | 98.9 | 94.1 | 94.8 | NA |
| Armenia | 6,376 | 99.8 | 98.7 | 86.9 | 78 | 39-49 |
| Urban China | NA | >95 | 95 | 100 | 83.3 | 81.8 |
| Urban IN | 10,713 | 97 | 87.9 | 91.1 | 46.9 | 17.3 |
| Urban BRA | 24,093 | 99.8 | 95.9 | NA | 94.9 | 49.3 |
| Urban ZAF | 25,149 | 91.7 | 84.0 | 92.1 | 78.7 | 44.1 |
